# Efficacy of treatment with corticosteroids for fibrotic hypersensitivity pneumonitis: a propensity score-matched cohort analysis

**DOI:** 10.1186/s12890-021-01608-1

**Published:** 2021-07-19

**Authors:** Masaru Ejima, Tsukasa Okamoto, Takafumi Suzuki, Tatsuhiko Anzai, Kunihiko Takahashi, Yasunari Miyazaki

**Affiliations:** 1 Department of Respiratory Medicine, Tokyo Medical and Dental University, 1-5-45 Yushima, Bunkyo-ku, Tokyo, 113-8519 Japan; 2 Department of Biostatistics M&D Data Science Center, Tokyo Medical and Dental University, Tokyo, Japan

**Keywords:** Fibrotic hypersensitivity pneumonitis, Propensity score matching, Corticosteroid

## Abstract

**Background:**

Fibrotic hypersensitivity pneumonitis (HP) is a chronic interstitial lung disease caused by allergic responses to repeated exposures to a causative antigen. Therapeutic evidence of the use of corticosteroids to treat fibrotic HP remains lacking, although corticosteroids are recognized as a major treatment option. The purpose of this study was to evaluate the efficacy of corticosteroid treatment in patients with fibrotic HP in a propensity score-matched cohort.

**Methods:**

A retrospective review of the medical records from 2005 to 2019 in a single center was conducted, and 144 patients with fibrotic HP were identified. Semiquantitative scores for lung abnormalities on HRCT were evaluated. Patients who received (PDN group) and did not receive (non-PDN group) corticosteroid treatment were matched using a propensity score method. Survival rates, serial changes in pulmonary function and annual changes in HRCT scores were compared in the matched cohort.

**Results:**

In the matched analysis, 30 individuals in the PDN group were matched with 30 individuals in the non-PDN group, the majority of whom had ILD without extensive fibrosis. The survival rate was significantly better in the PDN group (*P* = 0.032 for the stratified Cox proportional hazards model; HR, 0.250). The absolute changes in FVC at 6, 12, and 24 months from baseline were significantly better in the PDN group. Fewer patients in the PDN group experienced annual deterioration, as reflected in the HRCT score, due to ground-glass attenuation, consolidation, reticulation, traction bronchiectasis and honeycombing.

**Conclusion:**

We demonstrated that corticosteroids improved survival and slowed fibrotic progression in a matched cohort, the majority of whom had ILD without extensive fibrosis. Fibrotic HP with less severe fibrosis may benefit from corticosteroid treatment. We propose that the early initiation of corticosteroids should be considered for fibrotic HP when worsening fibrosis is observed.

**Supplementary Information:**

The online version contains supplementary material available at 10.1186/s12890-021-01608-1.

## Background

Hypersensitivity pneumonitis (HP) is an immune-mediated lung disease caused by repeated exposure to a sensitized antigen. HP is traditionally classified as acute or chronic [1]. Currently, the classification is described more specifically as nonfibrotic HP or fibrotic HP, given the clinical-radiologic-pathologic correlation that is linked to clinical outcomes [2]. Compared with nonfibrotic HP, which has a favorable prognosis, the clinical behavior of fibrotic HP may mimic that of idiopathic pulmonary fibrosis, resulting in high mortality [3, 4].

Corticosteroids serve as the main pharmacological treatment for fibrotic HP. Given the pathogenesis of HP as an immune-mediated disease, corticosteroids as anti-inflammatory agents are a reasonable treatment option. However, the evidence guiding treatment for fibrotic HP with corticosteroids is still based on observational data and expert opinions [5]. The effectiveness of corticosteroids has been reported only for the treatment of acute farmer’s lung [6]. A recent report regarding the effectiveness of corticosteroids for the treatment of fibrotic HP failed to demonstrate clinical benefits with regard to pulmonary function and survival in patients treated with corticosteroids compared with untreated patients [7]. In that study, differences in the underlying characteristics between the treated and untreated groups may have affected the comparison of treatment outcomes. It remains unclear whether corticosteroids change the clinical course of fibrotic HP.

Therefore, this study was conducted to demonstrate the efficacy of treatment for fibrotic HP with corticosteroids by matching the baseline characteristics of patients treated and not treated with corticosteroids and comparing the clinical outcomes. We aimed to identify the patients with fibrotic HP who could benefit from treatment with corticosteroids.

## Methods

### Patient selection and data collection

**Fig. 1:**
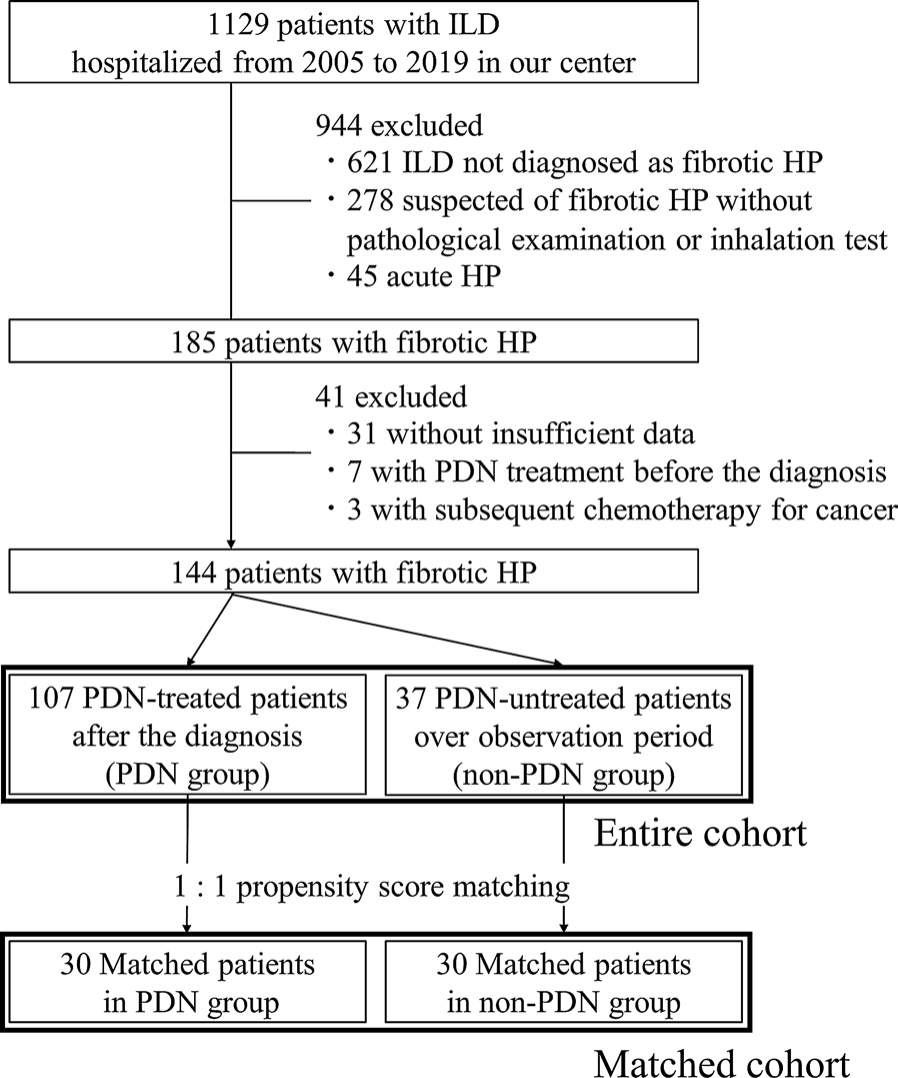
Patient recruitment flow diagram detailing the inclusion and exclusion of patients and the reasons for exclusion. The bold lines represent the patient groups included in further analysis. *HP* hypersensitivity pneumonitis; *ILD* interstitial lung disease; *PDN* prednisolone

Retrospective medical record reviews of consecutive patients with interstitial lung disease (ILD) who were hospitalized in our center between January 2005 and December 2019 were conducted, and 144 patients with fibrotic HP were identified. The patient recruitment flow diagram is shown in Fig. 1. Our respiratory center, as a specialized ILD institution, has been conducting in-hospital assessments of ILD patients to examine the etiology based on bronchoalveolar lavage (BAL) and lung tissue analyses. Our medical records review was limited to hospitalized patients to ensure that we could obtain adequate data to evaluate the diagnosis of fibrotic HP. The diagnosis of HP was validated based on a positive result on an inhalation challenge test and/or the pathological analysis of surgical lung biopsy, autopsy or transbronchial lung biopsy, using Yoshizawa’s criteria [8–12]. The presence of fibrosis was based on high-resolution computed tomography (HRCT) findings, including reticulation, traction bronchiectasis, and honeycombing. The level of confidence in the diagnosis of fibrotic HP according to the guidelines from the American Thoracic Society was reviewed for all 144 patients [2], and the majority of patients in our cohort had diagnoses classified as moderate to definite confidence: definite, 51; high, 37; moderate, 42; low, 12; and not excluded, 2. In the entire cohort, 107 patients who received treatment with prednisolone (PDN) (PDN group) and the remaining 37 patients who did not receive such treatment (non-PDN group) were identified. Thirty individuals in the PDN group were matched with 30 in the non-PDN group using a propensity score-matching method. The present study conformed to the principles of the Declaration of Helsinki and was approved by the institutional review board at Tokyo Medical and Dental University (M2019-206). The need to obtain written informed consent was waived due to the retrospective nature of this study.

Baseline data were reviewed on either the date of the initiation of treatment with corticosteroids in the PDN group or the date of diagnosis in the non-PDN group, which corresponded to the date of the decision regarding the use of corticosteroids. The absolute decline in the percent predicted forced vital capacity (%FVC) 12 months before registration was extrapolated from the %FVC data retrieved 6–18 months prior to the registration date. The follow-up pulmonary function data collected were the absolute changes in %FVC at 6 ± 3 months, 12 ± 3 months, and 24 ± 3 months. The last observation carried forward method was used to replace missing FVC measurements for censored or dead patients. The observation period ended at the last visit in five years or in April 2020. The survival period was defined as the time from the date of the initiation of treatment with corticosteroids in the PDN group or the date of diagnosis in the non-PDN group until the date of death from any cause or the last day patients were known to be alive in the observation period.

Missing %FVC data were imputed using the last observation carried forward method for censored or dead patients as follows: 0 patients at 6 months, 14 patients (13%) at 12 months, and 24 patients (32%) at 24 months in the entire PDN group and 0 patients at 6 months, 2 patients (5%) at 12 months, and 7 patients (19%) at 24 months in the entire non-PDN group. Missing %FVC data were imputed in the matched cohort as follows: 0 patients at 6 months, 1 patient (3%) at 12 months, and 4 patients (13%) at 24 months in the matched PDN group and 0 patients at 6 months, 1 patient (3%) at 12 months, and 7 patients (23%) at 24 months in the matched non-PDN group.

### Radiographic assessment

Evaluations of HRCT findings at baseline and the 1-year follow-up were performed using the Dutka/Vasakova scoring system by grading four levels: 1) aortic arch, 2) carina, 3) maximum diameter of the right ventricle, and 4) top of the right diaphragmatic dome [13].

The HRCT findings were semiquantitatively graded as summarized in Additional file 1: Table S1 and Figure S1. The HRCT findings were interpreted on the basis of the recommendations of the Nomenclature Committee of the Fleischner Society [14]. The extent of ground-glass attenuation (GGA), consolidation, reticulation, and honeycombing was graded using a scoring system from 0 to 5 on the basis of Kazerooni’s method [15], with slight modifications. The extent of traction bronchiectasis was assessed using a score from 0 to 3 as previously described [16, 17]. Bronchiolectasis, which is predominantly localized more distally in the lung, often within 2–5 mm of the pleura, was excluded from bronchiectasis to more accurately score the extent of bronchiectasis in this study. The presence of these HRCT findings and mosaic attenuation were also recorded. The maximum score and the total score for GGA, consolidation, reticulation, honeycombing, and traction bronchiectasis were recorded. A maximum score in any of the lung areas was recorded to evaluate the severity of each HRCT finding. The total score was calculated by summing the scores in 8 areas for each HRCT finding, and differences in the scores between the PDN group and the non-PDN group were evaluated.

Two pulmonary specialists (T.S. and M.E.), who were blinded to the clinical data, independently scored the HRCT features and reached consensus on any mismatches. The correlation coefficients for interobserver variation, which were used to evaluate the concordance of the total and maximum scores for each HRCT finding between the readers, showed excellent agreement, with Spearman *r* values ranging from 0.841 to 0.940 (*P* < 0.001).

### Statistical analysis

The data were analyzed using EZR software (Saitama, Japan, version 2.6-1) [18]. Data are presented as the means (standard deviations [SDs]), medians with interquartile ranges (IQRs), or numbers of patients and percentages, as appropriate. All statistical analyses were two-sided, and *P*-values of 0.05 or less were considered statistically significant. The Bonferroni correction adjusted the *P*-values for multiple comparisons. The correlation coefficients for interobserver variation, which were used to evaluate the concordance of the total and maximum scores for each HRCT finding between readers, were obtained using Spearman's rank correlation (*r*). Cumulative rates of mortality were estimated using the Kaplan–Meier method and were compared using the Cox proportional hazard regression model or the stratified Cox proportional hazard regression model.

The propensity score (PS) used to match patients between the PDN and non-PDN treatment groups was estimated for each patient with a logistic regression model using baseline patient characteristics. The following characteristics of patients were included in the model: age, sex, smoking history, %FVC, percent predicted expiratory volume in one second (%FEV1), and presence of honeycombing, traction bronchiectasis and mosaic attenuation on HRCT. The presence of these HRCT findings was included because they were previously demonstrated to be prognostic factors [4, 19–22]. PS matching using a 1:1 nearest-neighbor method without replacement was performed between the PDN and non-PDN groups, with calipers of 0.25 of the standard deviation of the logit of the PS [23]. This yielded a c-statistic of 0.815, indicating an adequate ability to differentiate between the two groups. Additional file 1: Figure S2 illustrates the dot plot of the distribution of PSs in both groups.

## Results

### Baseline patient characteristics

The baseline characteristics were compared between the PDN group (n = 107) and the non-PDN group (n = 37), as shown in Table 1. Patients in the PDN group had worse pulmonary function, as reflected in a mean %FVC of 58.2% and a more rapid decline in %FVC before registration. All HP patients were instructed to avoid exposure to causative antigens. Corticosteroids were continuously administered from baseline over the observation period in all patients in the treatment group, with a mean initial dose of approximately 0.5 mg/kg daily and a physician-dependent tapering schedule. In the baseline HRCT findings (Table 2), traction bronchiectasis and honeycombing were more frequently present in the PDN group, and the PDN group also had higher maximum scores for GGA, reticulation, traction bronchiectasis and honeycombing. The baseline characteristics were compared after matching between the PDN group (n = 30) and the non-PDN group (n = 30) (Table 1). There were no significant differences in most baseline parameters, including pulmonary function, with a mean %FVC of approximately 70%, although the 12-month %FVC decline before registration was more rapid in the PDN group. In the HRCT analysis (Table 2), significant differences between the groups in the presence of HRCT findings and in the distribution of maximum scores in all HRCT findings disappeared after matching. Therefore, the baseline characteristics and HRCT scores were mostly adequately adjusted by PS matching.

**Table 1:** Baseline characteristics in the entire cohort and in the matched cohort

	Entire cohort			Matched cohort		
	PDN group	Non-PDN group	*P*-value	PDN group	Non-PDN group	*P*-value
	n = 107	n = 37		n = 30	n = 30	
Age, years	64 [58–69]	69 [61–73]	0.016	65 [59–72]	69 [61–72]	0.452
Sex, male	63 (59%)	23 (62%)	0.876	15 (50%)	18 (60%)	0.505
Body weight, kg	57 [49–68]	58 [49–69]	0.703	56 [47–69]	59 [47–70]	0.365
Smoking, pack years	7 [0–38]	20 [0–47]	0.575	10 [0–30]	16 [0–34]	0.696
Ever smoked	55 (51%)	21 (57%)	0.145	17 (57%)	17 (57%)	0.999
FVC, ml	1890 (640)	2280 (740)	0.003	2260 (700)	2180 (740)	0.484
%FVC, %predicted	58.2 (14.2)	71.0 (15.0)	< 0.001	71.5 (13.2)	67.6 (14.0)	0.217
< 50%	31 (29%)	3 (8%)	0.012	1 (3%)	3 (10%)	0.617
%FVC decline, %predicted						
12 months before registration	13.3 (9.5)	5.2 (5.7)	< 0.001	12.0 (6.2)	5.0 (5.3)	< 0.001
FEV1, ml	1630 (530)	1910 (580)	0.007	1880 (580)	1820 (560)	0.565
%FEV1, %predicted	64.0 (14.2)	77.4 (14.2)	< 0.001	76.3 (11.7)	73.7 (12.2)	0.270
DLco, %	44.5 (18.2)	56.2 (23.7)	0.005	56.7 (14.7)	54.3 (25.6)	0.658
Serum KL-6, U/mL	1540 [1100–2590]	1070 [740–1590]	0.004	1420 [980–1960]	1070 [750–1860]	0.589
Serum SP-D, ng/mL	276 [176–437]	243 [180–317]	0.171	225 [130340]	260 [200–340]	0.424
BAL lymphocyte, %	17 [8–39]	16 [5–39]	0.546	22.9 [10.9–41.5]	20.5 [5.5–29.8]	0.520
Antigen^†^						
Bird-related	78 (73%)	31 (84%)	0.495	24 (80%)	24 (80%)	0.999
Home-related	14 (13%)	3 (8%)		3 (10%)	3 (10%)	
Unknown	15 (14%)	3 (8%)		3 (10%)	3 (10%)	
Treatment						
Anti-fibrotic agent	14 (13%)	8 (22%)	0.288	3 (10%)	7 (23%)	0.149
	PFD 11, NTD 3	PFD 4, NTD 4		PFD 1, NTD 2	PFD 3, NTD 4	
Immunosuppressant	49 (46%)	0	–	10 (33%)	0	–
	CYA 33, CYC 5, TAC 11			CYC 5, CyA 4, TAC 1		

Values are given as the number (percentage), mean (SD), or median [25th and 75th percentiles]. *P*-values were obtained from a two-sided Mann–Whitney U test, a chi-square test, or Fisher’s exact test for the comparison between the PDN group and the non-PDN group in the entire cohort. *P*-values were obtained from McNemar’s test, the Wilcoxon signed-rank test, a paired t-test, or Fisher’s exact test for the comparison between the PDN group and the non-PDN group in the matched cohort. *BAL* bronchoalveolar lavage; *BW* body weight; *CyA* cyclosporine A; *CYC* cyclophosphamide; *DLco* diffusion capacity of the lungs for carbon monoxide; *FEV1* forced expiratory volume in one second; *FVC* forced vital capacity; *HP* hypersensitivity pneumonitis; *KL-6* Krebs von den Lungen-6; *NTD* nintedanib; *PDN* prednisolone; *PFD* pirfenidone; *SD* standard deviation; *SP-D* surfactant protein-D; *TAC* tacrolimus

^†^The causative antigens were deemed bird-related when an inhalation test using an avian antigen and/or test for a serum antibody to an avian antigen was positive or home-related when an environmental challenge test or test for a serum anti-*Trichosporon asahii* antibody was positive

**Table 2:** Analysis of baseline HRCT findings in the entire cohort and in the matched cohort

Baseline HRCT findings	Entire cohort			Matched cohort		
	PDN group	Non-PDN group	*P*-value	PDN group	Non-PDN group	*P*-value
	n = 107	n = 37		n = 30	n = 30	
Presence of findings						
GGA	107 (100%)	36 (97%)	0.257	30 (100%)	30 (100%)	–
Consolidation	83 (78%)	29 (78%)	0.999	19 (63%)	23 (77%)	0.386
Reticulation	107 (100%)	36 (97%)	0.257	30 (100%)	30 (100%)	–
Traction bronchiectasis	99 (93%)	29 (78%)	0.030	24 (80%)	24 (80%)	0.999
Honeycombing	62 (58%)	14 (38%)	0.055	12 (40%)	12 (40%)	0.999
Mosaic attenuation	60 (56%)	16 (43%)	0.247	17 (57%)	14 (47%)	0.579
Maximum score, mean						
GGA	2.6	2.2	0.032	2.6	2.2	0.162
0/1/2/3/4/5 [%]	0/5/46/37/10/2	3/19/46/19/11/3	0.021	0/3/53/27/13/3	0/20/53/13/10/3	0.253
Consolidation	1.2	1.1	0.456	1.0	1.1	0.837
0/1/2/3/4/5 [%]	22/42/29/5/2/0	22/54/19/3/3/0	0.675	37/33/23/3/3/0	23/50/20/3/3/0	0.732
Reticulation	2.3	1.9	0.005	2.1	1.9	0.307
0/1/2/3/4/5 [%]	0/10/51/35/4/0	3/19/62/16/0/0	< 0.001	0/20/53/27/0/0	0/23/63/13/0/0	0.521
Traction bronchiectasis	1.6	1.1	0.001	1.1	1.1	0.878
0/1/2/3 [%]	7/40/42/10	22/51/27/0	< 0.001	20/47/33/0	20/50/30/0	0.999
Honeycombing	1.7	1.4	0.016	1.4	1.4	0.999
0/1/2/3/4/5 [%]	1/40/48/10/1/0	3/59/35/3/0/0	0.012	3/57/33/7/0/0	0/60/37/3/0/0	0.999

Values are given as numbers (percentages). *P*-values were obtained from a two-sided Mann–Whitney U test, a chi-square test, or Fisher’s exact test for the comparison between the PDN group and the non-PDN group in the entire cohort. *P*-values were obtained from McNemar’s test, the Wilcoxon signed-rank test, a paired t-test, or Fisher’s exact test for the comparison between the PDN group and the non-PDN group in the matched cohort. *HP* hypersensitivity pneumonitis; *PDN* prednisolone; *PFD* pirfenidone; *SD* standard deviation

ILD with severe fibrotic progression was more common in the entire cohort than the matched cohort, which mainly consisted of patients with ILD with mild to moderate fibrotic progression. Indeed, 29% of the patients in the entire cohort had a reduced %FVC less than 50%, which is the cutoff value for the worst physiology score in the ILD-GAP model [24]. The mean %FVC was 58.2%. In contrast, merely 3% of the patients in the matched cohort had a %FVC less than 50%, with a mean %FVC of approximately 70%. In addition, the distribution of maximum scores revealed that none of the patients in the matched cohort had a maximum reticulation score > 3 (25- < 50% in any of the lung areas) or a maximum traction bronchiectasis score = 3 (severe).

### Therapeutic effect on clinical outcomes

**Fig. 2:**
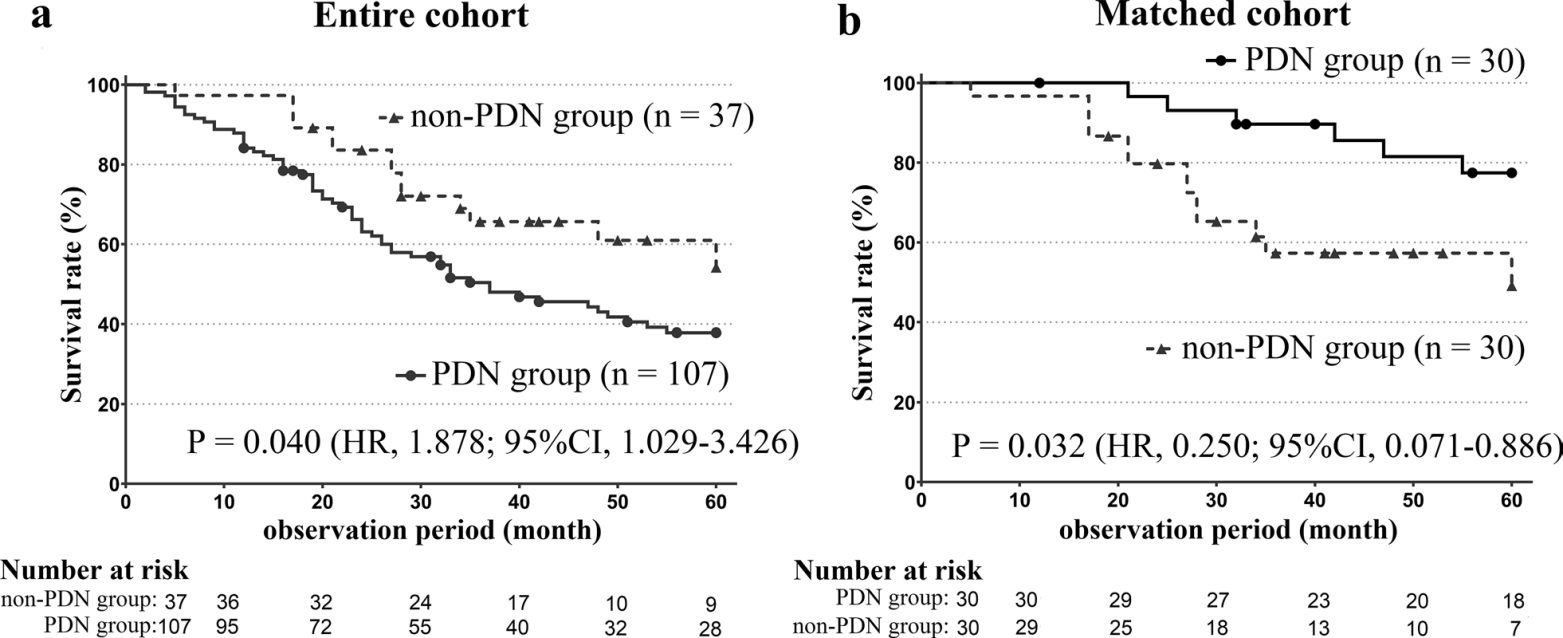
Kaplan–Meier curves for survival in the entire cohort and the matched cohort. Solid and dotted lines represent the PDN group and the non-PDN group, respectively. **a** In the entire cohort, the survival rate was significantly worse in the PDN group, with *P* = 0.040 (hazard ratio [HR], 1.878; 95% confidence interval [CI], 1.029–3.426). The median survival periods were 37 months (95% CI 26–55 months) and NR, respectively. **b** In the matched cohort, the survival rate was better in the PDN group, with *P* = 0.032 (HR, 0.250; 95% CI, 0.071–0.886). The median survival durations were NR and 60 months, respectively. *CI* confidence interval; *HR* hazard ratio; *NR* not reached; *PDN* prednisolone; *SD* standard deviation

Figure 2 shows the Kaplan–Meier curves for survival in the PDN group and the non-PDN group. In the entire cohort (Fig. 2a), the survival rate was significantly worse in the PDN group, with *P* = 0.040 (hazard ratio [HR], 1.878; 95% confidence interval [CI], 1.029–3.426). Conversely, in the matched cohort (Fig. 2b), the survival rate was better in the PDN group, with *P* = 0.032 (HR, 0.250; 95% CI, 0.071–0.886). This result demonstrated that treatment with corticosteroids improved the survival rate in the matched cohort.

**Fig. 3:**
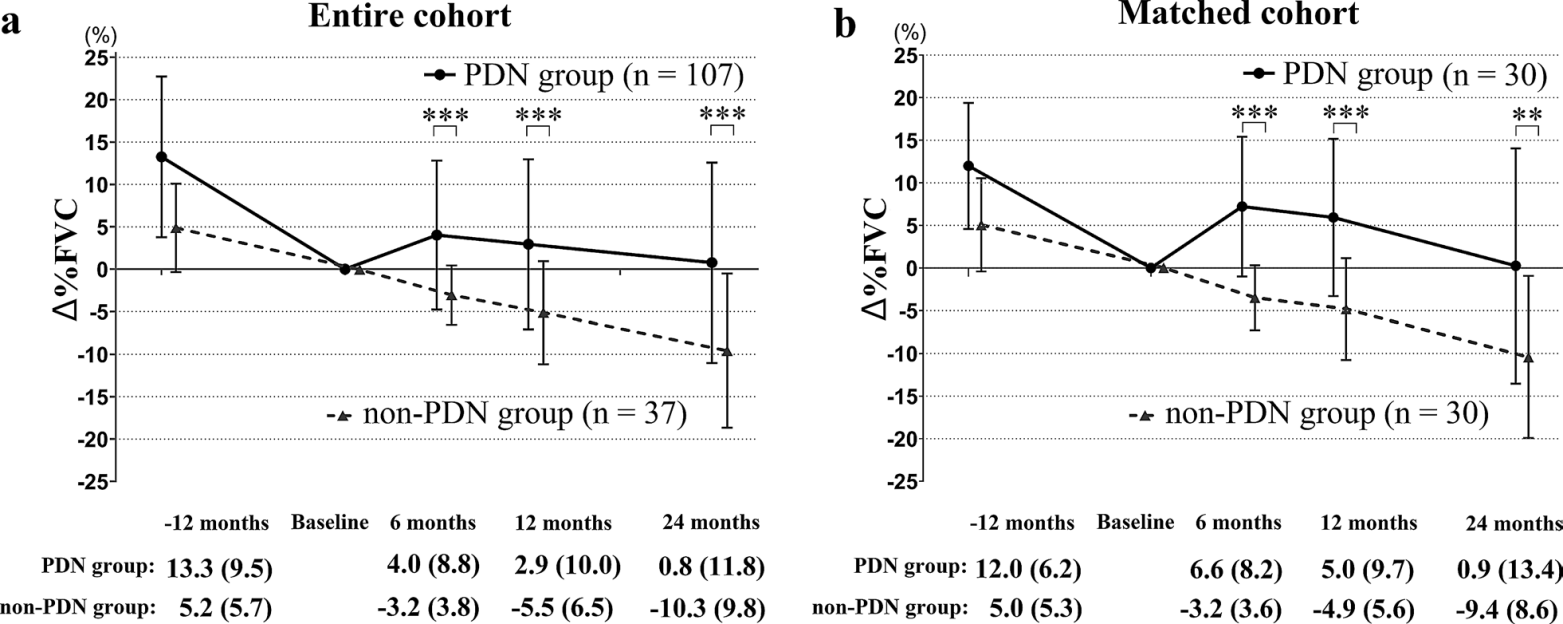
Absolute changes in %FVC from baseline in the entire cohort and the matched cohort. Between-group differences in the absolute changes in %FVC from baseline (Δ%FVC) described as the mean (SD) at the 6-, 12-, and 24-month follow-ups were compared. Solid and dotted lines represent the PDN group and the non-PDN group, respectively. **a** In the entire cohort, the Δ%FVC in the PDN group was significantly different from that in the non-PDN group at 6 months (4.0% [8.8] vs − 3.2% [3.8], *P* < 0.001), 12 months (2.9% [10.0] vs − 5.5% [6.5], *P* < 0.001), and 24 months (0.8% [11.8] vs − 10.3% [9.8], *P* < 0.001). P-values were obtained with the Mann–Whitney U test. **b** In the matched cohort, the Δ%FVC in the PDN group was significantly different from that in the non-PDN group at 6 months (6.6% [8.2] vs − 3.2% [3.6], *P* < 0.001), 12 months (5.0% [9.7] vs − 4.9% [5.6], P < 0.001), and 24 months (0.9% [13.4] vs − 9.4% [8.6], *P* = 0.001). *P*-values were obtained with the Wilcoxon signed-rank test. A *** indicates a *P*-value < 0.001, a ** indicates a *P*-value < 0.01. *FVC* forced vital capacity; *PDN* prednisolone; *SD* standard deviation

Figure 3 shows the comparison of the absolute change in %FVC from baseline (Δ%FVC) between the PDN group and the non-PDN group. In the entire cohort (Fig. 3a), the Δ%FVC in the PDN group was significantly different from that in the non-PDN group at 6 months (4.0% vs − 3.2%, *P* < 0.001), 12 months (2.9% vs − 5.5%, *P* < 0.001), and 24 months (0.8% vs − 10.3%, *P* < 0.001). Similarly, in the matched cohort (Fig. 3b), the Δ%FVCs in the PDN group was significantly different from that in the non-PDN group at 6 months (6.6% vs − 3.2%, *P* < 0.001), 12 months (5.0% vs − 4.9%, *P* < 0.001), and 24 months (0.9% vs − 9.4%, *P* = 0.001). These results demonstrated that treatment with corticosteroids improved pulmonary function over the course of two years in our cohort.

**Fig. 4:**
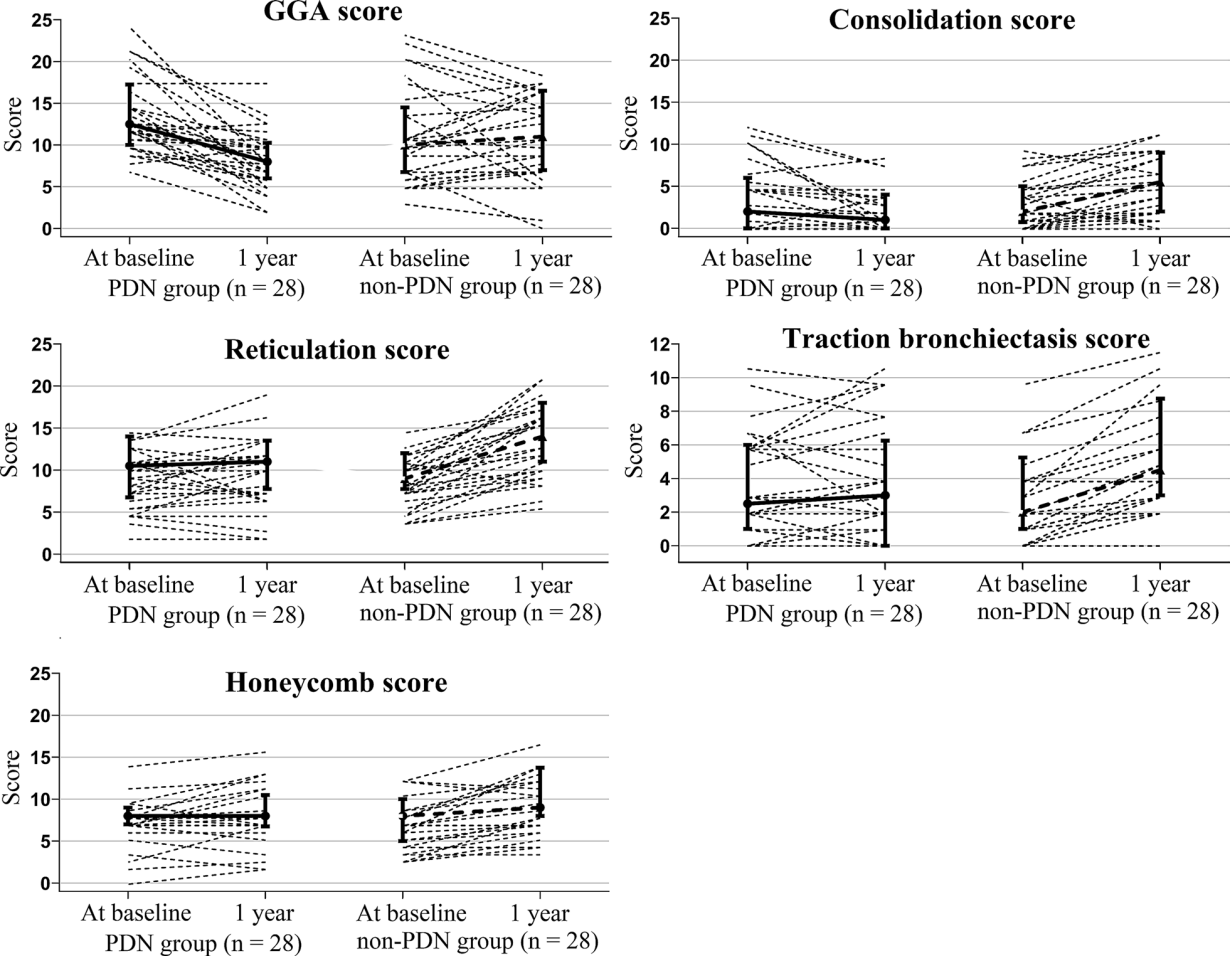
Trends in the 1-year change in total scores from baseline for each HRCT finding. The total scores for GGA and consolidation appeared to improve, with slow progression in reticulation, traction bronchiectasis, and honeycombing in the PDN group. The whiskers at the bottom and top represent the 25th and 75th percentiles, respectively. The middle horizontal lines are the median. *GGA* ground glass attenuation; *PDN* prednisolone

Table 3 and Fig. 4 describe the changes in total scores from baseline to 1 year in the matched cohort. As shown in Table 3, significantly fewer patients in the PDN group experienced deteriorations in the scores for all HRCT findings. As shown in Fig. 3, the scores in the PDN group appeared to improve for GGA and consolidation, and the progression in reticulation, traction bronchiectasis, and honeycombing was slow. In contrast, the scores for all HRCT findings appeared to worsen in the non-PDN group. These results indicated that treatment with corticosteroids improved the acute inflammatory findings of GGA and consolidation and inhibited the progression of the fibrotic components of reticulation, traction bronchiectasis, and honeycombing in our cohort.

**Table 3:** Total HRCT scores in the matched cohort

Matched cohort	PDN group	Non-PDN group	*P*-value
Total HRCT scores (baseline)	n = 30	n = 30	
GGA	13 [10–17]	10 [7–14]	0.033
Consolidation	2 [0–6]	2 [1–5]	0.593
Reticulation	11 [7–14]	9 [8–12]	0.592
Traction bronchiectasis	3 [1–6]	2 [1–5]	0.614
Honeycombing	8 [7–9]	8 [5–10]	0.779
Total HRCT scores (1-year follow-up)	n = 28	n = 28	
GGA	8 [6–10]	11 [7–16]	0.031
Consolidation	1 [0–4]	6 [2–9]	0.005
Reticulation	11 [8–14]	14 [11–18]	0.018
Traction bronchiectasis	3 [0–6]	5 [3–8]	0.077
Honeycombing	8 [7–11]	9 [8–13]	0.166
1-year change in total scores, n (deterioration/no deterioration)^†^	n = 28	n = 28	
GGA	(2/26)	(15/13)	< 0.001
Consolidation	(4/24)	(21/7)	< 0.001
Reticulation	(12/16)	(27/1)	< 0.001
Traction bronchiectasis	(10/18)	(24/4)	0.001
Honeycombing	(8/20)	(19/9)	0.008

Total HRCT scores at baseline and the 1-year follow-up and the 1-year change in total scores in the matched cohort were calculated. HRCT data were obtained for 28 of the 30 patients at the 1-year follow-up. Values are given as numbers or medians [25th and 75th percentiles]. *P*-values were obtained from the Wilcoxon signed-rank test and McNemar’s test. *GGA* ground glass attenuation; *HRCT* high-resolution computed tomography; *PDN* prednisolone

^†^The 1-year change in total scores was evaluated dichotomously as indicating deterioration when a total score at the 1-year follow-up was increased from that at baseline or indicating no deterioration when the total score was the same or decreased

**Fig. 5:**
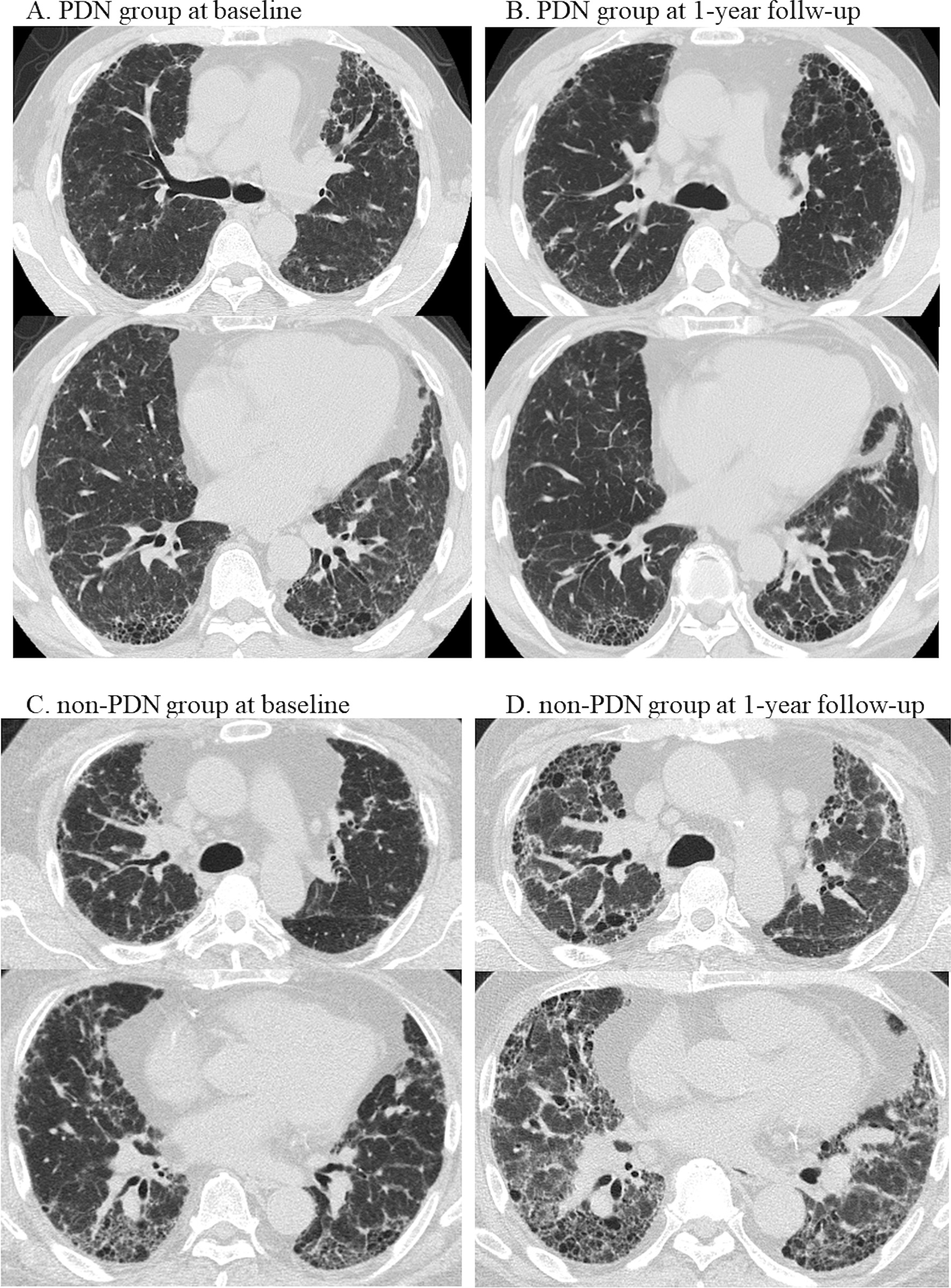
Representative matched pairs of patients. **A**, **B** A 63-year-old male with fibrotic bird-related hypersensitivity pneumonitis was treated with prednisolone from baseline until the end of the follow-up period at 60 months. At baseline, the patient’s %FVC was 57.9%, his annual %FVC decline before treatment was 26.1%, and his BAL lymphocyte count was 29%. At the 1-year follow-up, the change in %FVC from baseline was + 15.9%. A reduction in the extent of GGA with minimal fibrotic progression was observed on HRCT after one year. The total scores for the HRCT findings (baseline to the 1-year follow-up) were as follows: GGA (22 to 14), consolidation (2 to 0), reticulation (12 to 13), traction bronchiectasis (6 to 8), honeycombing (13 to 14), and mosaic attenuation (2 to 0). **C**, **D** A 66-year-old male with fibrotic bird-related hypersensitivity pneumonitis did not undergo treatment with corticosteroids and died at 17 months. At baseline, his %FVC was 46.6%, and his annual FVC decline until the date of diagnosis was 4.7%. At the 1-year follow-up, the change in %FVC from baseline was − 12.3%. Severe fibrotic progression was observed on HRCT. The total scores for the HRCT findings were as follows: GGA (11 to 18), consolidation (5 to 6), reticulation (16 to 19), traction bronchiectasis (10 to 15), honeycombing (14 to 19), and mosaic attenuation (0 to 0). *BAL* bronchoalveolar lavage; *%FVC* percent forced vital capacity; *GGA* ground glass attenuation; *HP* hypersensitivity pneumonitis; *HRCT* high-resolution computed tomography

Representative matched pairs are shown in Fig. 5.

## Discussion

Our study aimed to provide evidence supporting treatment with corticosteroids for fibrotic HP. We demonstrated that corticosteroids improved survival and inhibited radiologic fibrotic progression and %FVC decline in the matched cohort, which was primarily composed of patients with ILD without extensive fibrosis. Our analyses suggest that the early initiation of treatment with corticosteroids may benefit patients with fibrotic HP.

This is the first report to demonstrate the survival benefit associated with treatment with corticosteroids in patients with fibrotic HP. The analysis showed better survival in the PDN group, after matching for pulmonary function and the radiologic presence of fibrosis, which are recognized as predictive of mortality. Indeed, reduced FVC is also recognized as a predictor of mortality in patients with fibrotic HP [25]. The presence of honeycombing is an established prognostic factor in patients with various fibrotic ILDs, including HP [4, 19, 22]. A previous report evaluating the effectiveness of corticosteroids for the treatment of fibrotic HP failed to demonstrate a survival benefit of corticosteroids in a cohort with a lower FVC and a relatively high frequency of the presence of honeycombing in the treatment group [7]. Consistent with this finding, the PDN group in our entire cohort, which similarly consisted of more patients with advanced progression of ILD, with a lower FVC and more radiologic evidence of fibrosis, had a worse survival rate than the group that was not treated with PDN. These differences in underlying characteristics between the groups seemed to lead to the underestimation of the effectiveness of treatment. Notably, the matched cohort consisted of many patients with earlier stages of ILD, with lower scores for fibrosis. Therefore, our results may be applicable only to patients with ILD without extensive fibrosis. Whether corticosteroids yield therapeutic benefits for HP patients with extensive fibrosis remains unclear.

Regarding the radiographic assessment, the scores for the fibrotic components of reticulation, traction bronchiectasis and honeycombing were surprisingly stable in the PDN group; in addition, the scores for the acute inflammatory findings of GGA and consolidation improved in the PDN group. In contrast, all these components worsened in most patients in the non-PDN group. This result indicates that corticosteroids may slow the progression of fibrosis, which is feasible, given that immune-mediated inflammation in HP is a target of anti-inflammatory medications. There have been several reports of the reversal of traction bronchiectasis and reticulation with treatment, although these fibrotic components typically worsen over time. In an analysis of nonspecific interstitial pneumonia that compared the extent of HRCT findings between the initial and later timepoints, some instances of traction bronchiectasis in the early stage were reversed, which was presumably attributable to the collapse of the surrounding peripheral lung parenchyma [26]. In addition, reticulation, which is primarily associated with fibrosis, was slightly improved by pharmacological therapy in some patients [27]. It is possible that the progression of these fibrotic components could be slowed by treatment with corticosteroids, which was supported by our findings regarding the annual changes in HRCT scores. These observations imply that treatment with corticosteroids may benefit patients in the early stages of fibrotic HP due to its inhibitory effect on fibrotic progression and ameliorating effect on acute inflammation.

With respect to the effect of treatment on pulmonary function, our results showed that treatment with corticosteroids improved pulmonary function over the course of two years. It is plausible that the reduction in the extent of GGA led to an improvement in pulmonary function in our cohort, as the scores for GGA decreased after the initiation of corticosteroids in many patients. As reported previously, the resolution of GGA primarily reflects lymphocytic inflammation that is linked to an improvement in pulmonary function in patients with fibrosing lung diseases [28, 29]. Meanwhile, our result was inconsistent with a previous report, which showed no effect of treatment with corticosteroids in patients with fibrotic HP [7]. The inconsistency may be attributed to the duration of the treatment period, as the mean treatment duration was half a year in that previous study, while corticosteroids were used continuously over the observation period in our study. Whether a response of pulmonary function to treatment leads to a survival benefit remains unclear, given that the PDN group in the entire cohort also experienced a posttreatment improvement in FVC, although the mortality rate was high. This clinical question needs to be investigated further.

Evidence supporting the benefits of treatment with corticosteroids for fibrotic HP has been lacking thus far. Given that HP is an immune-mediated disease, treatment with antiinflammatory agents such as corticosteroids may be a reasonable option even when fibrotic lesions are dominant findings on HRCT. We demonstrated that the PDN group in the matched cohort of patients with mild to moderate fibrosis had improved survival and slower radiologic fibrotic progression, while in the entire cohort, including the population with advanced fibrotic progression, the PDN group did not have better survival. Therefore, treatment with corticosteroids may need to be initiated before fibrosis becomes advanced. Mild to moderate fibrosis was identified as a therapeutic target based on the baseline variables of %FVC > 50%, maximum reticulation score < 4, and maximum traction bronchiectasis score < 3 in the majority of the population in our matched cohort. In addition, the population with extensive GGA, which is suggestive of a more abundant inflammatory process, may benefit from treatment with corticosteroids, as might patients with acute inflammatory HP. In our matched analysis, the total GGA score decreased dramatically after treatment, which may have been related to the improved outcomes. In addition, the presence of GGA was associated with improved survival in a multivariate model with adjustment for honeycombing in patients with chronic HP [30]. A recent review proposed that signs of active inflammation, including extensive GGA, were indications for the initiation of immunosuppressive therapy in fibrotic HP patients [5]. Based on this evidence, we suggest that patients with HP with mild to moderate fibrosis and evidence of GGA could be a promising target population for treatment with corticosteroids.

With regard to the pharmacological options for the treatment fibrotic HP, whether antifibrotic agents or immunosuppressants, including corticosteroids and other agents, should be selected remains to be discussed. There have been no head-to-head comparison studies between these interventions yet, and it would be interesting to conduct such studies in the future. In the latest report, nintedanib, a major antifibrotic agent, reduced the rate of ILD progression in patients with chronic fibrosing ILDs with progressive phenotypes, including patients with fibrotic HP [31, 32]. However, the primary outcome of that study focused on pulmonary function decline instead of survival, which was not statistically identified in the analysis of secondary outcome. In contrast, another previous study reported that mycophenolate mofetil or azathioprine for the management of chronic HP failed to yield positive outcomes with regard to pulmonary function and survival [33]. In our cohort, 33% of the group treated with corticosteroids was subsequently treated with other immunosuppressive agents, which could have contributed to the observed clinical improvement. We believe that the corticosteroids were the cause of the favorable outcomes in our analysis. However, intensive immunosuppressive therapy using corticosteroids and other immunosuppressants as a first-line option may be worth examining.

This study has several limitations. First, the matched cohort was small. This is mainly because the patients with progressed ILD were mostly receiving a therapeutic intervention, and we could not collect more patients for inclusion in the non-PDN group. Therefore, the results in the present study need to be verified in a larger cohort before being broadly applied. Second, it remains unclear whether the unmatched patients in this study who had severe disease and a high mortality rate benefits from corticosteroids. Exploring different matching strategies and outcomes may help overcome these limitations. Third, a quantitative scoring system was not adopted in this study, although such systems have been commonly used to more precisely estimate the extent of HRCT abnormalities. Instead, we used a semiquantitative assessment due to the ease of measurement and utility in clinical practice. Finally, the optimal dose and duration of therapy and the risk of adverse events were not discussed in this study. The mean initial dosage of corticosteroids in our cohort was approximately 0.5 mg/kg daily on an individual tapering schedule without complete withdrawal over the clinical course, which resulted in improved survival in the matched cohort. Whether a lower dose of corticosteroids results in a different outcome may be a question of interest with respect to the need to minimize the long-term adverse effects of therapy.

## Conclusions

This single-center study demonstrated that corticosteroids may favorably alter the clinical course of fibrotic HP in a population without extensive fibrosis. Patients with an early stage of fibrotic HP with observed fibrotic progression may benefit from treatment with corticosteroids in terms of survival and fibrotic progression. We believe these findings will serve as a guide for the initiation of treatment with corticosteroids in clinical practice.

## Supplementary Information

**Additional file 1: Figures S1, S2 and Table S1**. Figure S1 Semiquantitative scoring system for traction bronchiectasis used in this study. Figure S2 A dot plot of the distribution of propensity scores in the PDN group and the non-PDN group. Table S1 Summary of the semiquantitative scoring system for HRCT findings.

## Data Availability

The datasets analyzed during the current study are available from the corresponding author on reasonable request.

## Abbreviations

BAL: Bronchoalveolar lavage
BW: Body weight
CI: Confidence interval
CyA: Cyclosporine A
CYC: Cyclophosphamide
DLco: Diffusion capacity of the lungs for carbon monoxide
FEV1: Forced expiratory volume in one second
FVC: Forced vital capacity
GGA: Ground glass attenuation
HP: Hypersensitivity pneumonitis
HR: Hazard ratio
HRCT: High-resolution computed tomography
ILD: Interstitial lung disease
KL-6: Krebs von den Lungen-6
NR: Not reached
NTD: Nintedanib
PDN: Prednisolone
PFD: Pirfenidone
PS: Propensity score
SD: Standard deviation
SP-D: Surfactant protein-D
TAC: Tacrolimus
