# Supplementary material for: Efficacy of treatment with corticosteroids for fibrotic hypersensitivity pneumonitis: a propensity score-matched cohort analysis
Source: BMC Pulm Med. 2021 Jul 19;21:243. doi: 10.1186/s12890-021-01608-1 (PMC8290597; doi:10.1186/s12890-021-01608-1)
Supplement: Supplementary file 1 — Additional file 1: Figures S1, S2 and Table S1. Figure S1 Semiquantitative scoring system for traction bronchiectasis used in this study. Figure S2 A dot plot of the distribution of propensity scores in the PDN group and the non-PDN group. Table S1 Summary of the semiquantitative scoring system for HRCT findings. [file 12890_2021_1608_MOESM1_ESM.pdf]

Supplementary appendix

Figure S1- Semiquantitative scoring for traction bronchiectasis used in this study.

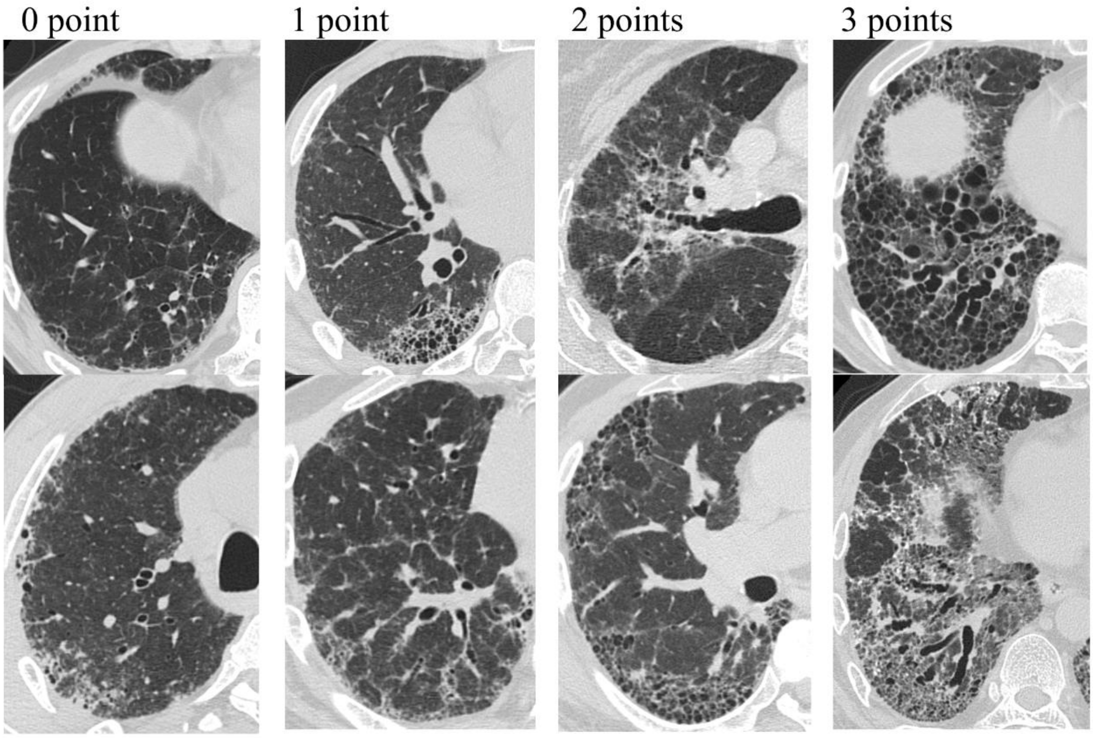

0 point = absent, 1 point = minor (single scattered changes), 2 points = moderate (larger single changes or several minor changes), 3 points = severe (uniform or substantial changes).

Figure S2- A dot plot of the distribution of propensity scores in the PDN group and the non-PDN group.

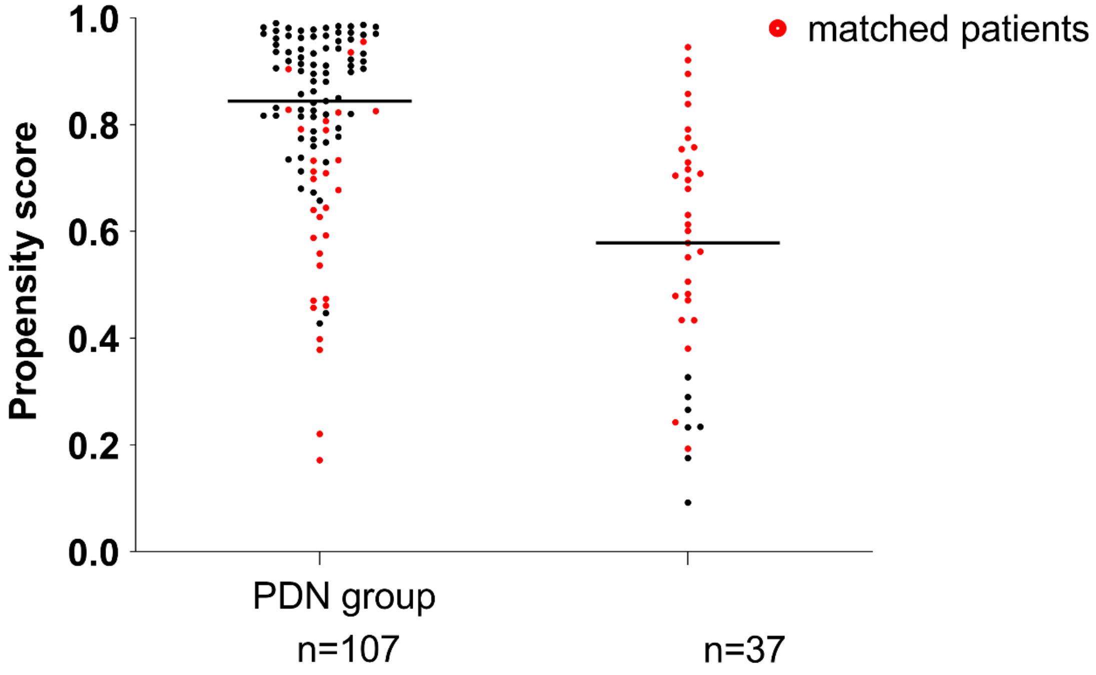

The middle horizontal line represents the mean. Matched patients are highlighted in red dots.

PDN: prednisolone.

Table S1- Summary of the semiquantitative scoring system for HRCT findings.

Findings (Total score)	Score for each of 8 areas
Ground-glass attenuation<br>(0-40)	0 = Absent<br>1 = Ground-glass attenuation involving <5% of the area (minimal, but not normal)<br>2 = Ground-glass attenuation involving 5-<25% of the area<br>3 = Ground-glass attenuation involving 25-<50% of the area<br>4 = Ground-glass attenuation involving 50-<75% of the area<br>5 = Ground-glass attenuation involving >75% of the area
Consolidation<br>(0-40)	0 = Absent<br>1 = Consolidation involving <5% of the area (minimal, but not normal)<br>2 = Consolidation involving 5-<25% of the area<br>3 = Consolidation involving 25-<50% of the area<br>4 = Consolidation involving 50-<75% of the area<br>5 = Consolidation involving >75% of the area
Reticulation<br>(0-40)	0 = Absent<br>1 = Reticulation involving <5% of the area (minimal, but not normal)<br>2 = Reticulation involving 5-<25% of the area<br>3 = Reticulation involving 25-<50% of the area<br>4 = Reticulation involving 50-<75% of the area<br>5 = Reticulation involving >75% of the area
Honeycombing<br>(0-40)	0 = Absent<br>1 = Interlobular septal thickening; no discrete honeycombing<br>2 = Honeycombing (with or without septal thickening) involving <25% of the area<br>3 = Honeycombing (with or without septal thickening) involving 25-<50% of the area<br>4 = Honeycombing (with or without septal thickening) involving 50-<75% of the area<br>5 = Honeycombing (with or without septal thickening) involving >75% of the area
Traction bronchiectasis<br>(0-24)	0 = Absent<br>1 = Mild (Single scattered changes)<br>2 = Moderate (Larger single changes or several minor changes)<br>3 = Severe (Uniform or substantial changes)
